# Adenosine Signaling through A1 Receptors Inhibits Chemosensitive Neurons in the Retrotrapezoid Nucleus

**DOI:** 10.1523/ENEURO.0404-18.2018

**Published:** 2018-12-14

**Authors:** S. D. James, V. E. Hawkins, B. Falquetto, D. N. Ruskin, S. A. Masino, T. S. Moreira, M. L. Olsen, D. K. Mulkey

**Affiliations:** 1 Department of Physiology and Neurobiology, University of Connecticut, Storrs, CT; 2 Department of Pharmacology, Institute of Biomedical Sciences, University of São Paulo, São Paulo, Brazil; 3 Neuroscience Program, Department of Psychology, Trinity College Hartford, CT; 4 Department of Physiology and Biophysics, Institute of Biomedical Sciences, University of São Paulo, São Paulo, Brazil; 5 School of Neuroscience, Virginia Polytechnic Institute and State University, Blacksburg, VA

**Keywords:** brain slice, cellular, chemoreception, network, RTN

## Abstract

A subset of neurons in the retrotrapezoid nucleus (RTN) function as respiratory chemoreceptors by regulating depth and frequency of breathing in response to changes in tissue CO_2_/H^+^. The activity of chemosensitive RTN neurons is also subject to modulation by CO_2_/H^+^-dependent purinergic signaling. However, mechanisms contributing to purinergic regulation of RTN chemoreceptors are not entirely clear. Recent evidence suggests adenosine inhibits RTN chemoreception *in vivo* by activation of A1 receptors. The goal of this study was to characterize effects of adenosine on chemosensitive RTN neurons and identify intrinsic and synaptic mechanisms underlying this response. Cell-attached recordings from RTN chemoreceptors in slices from rat or wild-type mouse pups (mixed sex) show that exposure to adenosine (1 µM) inhibits chemoreceptor activity by an A1 receptor-dependent mechanism. However, exposure to a selective A1 receptor antagonist (8-cyclopentyl-1,3-dipropylxanthine, DPCPX; 30 nM) alone did not potentiate CO_2_/H^+^-stimulated activity, suggesting activation of A1 receptors does not limit chemoreceptor activity under these reduced conditions. Whole-cell voltage-clamp from chemosensitive RTN neurons shows that exposure to adenosine activated an inward rectifying K^+^ conductance, and at the network level, adenosine preferentially decreased frequency of EPSCs but not IPSCs. These results show that adenosine activation of A1 receptors inhibits chemosensitive RTN neurons by direct activation of a G-protein-regulated inward-rectifier K^+^ (GIRK)-like conductance, and presynaptically, by suppression of excitatory synaptic input to chemoreceptors.

## Visual Abstract

**Figure:**
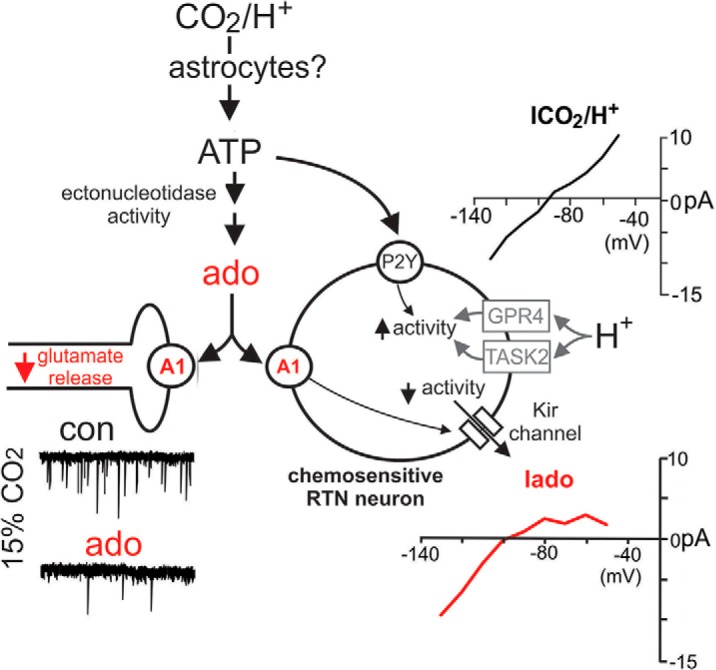

## Significance Statement

Adenosine is a potent modulator of all aspects of breathing including chemoreception at the level of the retrotrapezoid nucleus (RTN); however, mechanisms by which adenosine regulates activity of RTN chemoreceptors is not known. Here, we show that adenosine activation of A1 receptors inhibits RTN neurons by activation of an inward rectifying K^+^ conductance, and by selective suppression of excitatory synaptic input to chemoreceptors. These results identify a G-protein-regulated inward-rectifier K^+^ (GIRK)-like conductance as the first target of purinergic signaling in chemosensitive RTN neurons. This work may also have clinical relevance since A1 receptor antagonists like caffeine are used to treat respiratory problems in premature infancy.

## Introduction

Central chemoreception is the mechanism by which the brain senses changes in tissue CO_2_/H^+^ to regulate breathing (Nattie and Li, 2012). A brainstem region called the retrotrapezoid nucleus (RTN) is an important site of chemoreception (Guyenet and Bayliss, 2015; Guyenet et al., 2016). Neurons in this region are intrinsically sensitive to H^+^ (Wang et al., 2013) and possibly HCO_3_^–^ (Goncalves and Mulkey, 2018); however, their activity is also subject to modulation by various transmitters including CO_2_/H^+^-evoked ATP release presumably from local chemosensitive astrocytes. For example, ATP-purinergic signaling through P2Y receptors has been shown to activate RTN neurons directly (Mulkey et al., 2006; Gourine et al., 2010; Wenker et al., 2012; Barna et al., 2016) and indirectly by mediating vasoconstriction to maintain tissue CO_2_/H^+^ (Hawkins et al., 2017). However, extracellular ATP can be rapidly metabolized to adenosine (Dunwiddie and Masino, 2001) which then may serve to counterbalance the excitatory effects of P2 signaling by suppressing CO_2_/H^+^-dependent output of the RTN in both awake and anesthetized rats (Falquetto et al., 2018). This possibility is consistent with the hypothesis that adenosine signaling through A1 receptors functions as a braking mechanism during times of high chemoreceptor drive (Montandon et al., 2008). Also, perhaps not surprisingly, adenosine inhibition of RTN chemoreception *in vivo* was shown to involve A1 receptors (Falquetto et al., 2018) which are highly expressed in the ventrolateral medulla near the RTN (Bissonnette and Reddington, 1991); however, the cellular and network basis for A1 receptor-dependent inhibition of RTN neurons remains unknown.

Adenosine A1 receptors are G_i_/G_o_-coupled and in other brain regions are known to inhibit neural activity by presynaptic and postsynaptic mechanisms. At the presynaptic level, activation of A1 receptors has been shown to suppress neurotransmitter release by cAMP-independent mechanisms involving inhibition of voltage gated Ca^2+^ channels (Cunha, 2001; Sebastião and Ribeiro, 2009). Interestingly, in the hippocampus (Lambert and Teyler, 1991; Yoon and Rothman, 1991), adenosine signaling through A1 receptors preferentially suppressed excitatory over inhibitory synaptic currents. Postsynaptically, A1 receptor activation can hyperpolarize membrane potential and inhibit neural activity by cAMP-dependent inhibition of HCN channels (Li et al., 2011) and βγ-subunit-dependent activation of G-protein-regulated inward-rectifier K^+^ (GIRK; Kir3) channels (Lüscher et al., 1997; Cunha, 2001; Dunwiddie and Masino, 2001). It should also be noted that A1 receptors can interact with other G-proteins as well as ionotropic receptors (Sichardt and Nieber, 2007) and so have the potential to influence neuronal excitability by a multitude of mechanisms. The main goal of this study was to characterize effects of adenosine on chemosensitive RTN neurons and identify intrinsic and synaptic mechanisms underlying this response.

Consistent with our previous *in vivo* results (Falquetto et al., 2018), we find at the level of the RTN that adenosine strongly inhibits activity of RTN neurons by an A1 receptor-dependent mechanism. We also show that mechanisms contributing to this response involve activation of an inward rectifying K^+^ conductance, and selective suppression of excitatory synaptic input to chemoreceptors. These results are consistent with known mechanisms by which adenosine and A1 receptors inhibits neural activity in other brain regions (Cunha, 2001; Dunwiddie and Masino, 2001). These results may be clinically relevant since they identify chemosensitive RTN neurons as potential cellular targets for the respiratory-stimulating effects of caffeine (D'Urzo et al., 1990; Pianosi et al., 1994), an A1 and A2 receptor antagonist used therapeutically to mitigate breathing problems in premature infants (Stevenson, 2007). Furthermore, these results also suggest that activation of A1 receptors as a treatment for controlling seizure activity in epilepsy (Etherington and Frenguelli, 2004) may suppress output of the RTN and increase the risk of respiratory problems, which are a leading cause of sudden unexpected death in epilepsy (SUDEP; Devinsky et al., 2016).

## Materials and Methods

### Animals

Animal use was in accordance with guidelines approved by the University of Connecticut and Trinity College Institutional Animal Care and Use Committee. The majority of *in vitro* experiments were performed using brain slices isolated from mixed sex neonatal Sprague Dawley rat pups (7–12 d old; *N* = 75 rats; Charles River Laboratories). A parallel series of *in vitro* experiments were performed in brain slices from age matched mixed sex C57/B6 mice (*N* = 6 mice; Jackson Laboratories). All efforts were made to minimize animal discomfort and the number of animals used.

### Electrophysiological recordings in brainstem slices

Slices containing the RTN were prepared as previously described (Kuo et al., 2016; Goncalves and Mulkey, 2018). In short, rats were anesthetized by administration of ketamine (375 mg/kg; i.p.) and xylazine (25 mg/kg; i.p.) and rapidly decapitated; brainstems were removed and transverse brainstem slices (300 μm) were cut using a microslicer (DSK 1500E; Dosaka) in ice-cold substituted Ringer solution containing the following: 260 mM sucrose, 3 mM KCl, 5 mM MgCl_2_, 1 mM CaCl_2_, 1.25 mM NaH_2_PO_4_, 26 mM NaHCO_3_, 10 mM glucose, and 1 mM kynurenic acid. Slices were incubated for 30 min at 37°C and subsequently at room temperature in a normal Ringer’s solution containing: 130 mM NaCl, 3 mM KCl, 2 mM MgCl_2_, 2 mM CaCl_2_, 1.25 mM NaH_2_PO_4_, 26 mM NaHCO_3_, and 10 mM glucose. Both substituted and normal Ringer’s solutions were bubbled with 95% O_2_ and 5% CO_2_ (pH 7.30).

Individual slices containing the RTN were transferred to a recording chamber mounted on a fixed-stage microscope (Olympus BX5.1WI) and perfused continuously (∼2 ml/min) with a bath solution containing: 140 mM NaCl, 3 mM KCl, 2 mM MgCl_2_, 2 mM CaCl_2_, 10 mM HEPES, and 10 mM glucose (equilibrated with 5% CO_2_; pH 7.3). All recordings were made with an Axopatch 200B patch-clamp amplifier, digitized with a Digidata 1322A A/D converter, and recorded using pCLAMP 10.0 software (Molecular Devices). Recordings were obtained at room temperature (∼22°C) with patch electrodes pulled from borosilicate glass capillaries (Harvard Apparatus) on a two-stage puller (P-97; Sutter Instrument) to a DC resistance of 5–7 MΩ when filled with a pipette solution containing the following: 120 mM KCH_3_SO_3_, 4 mM NaCl, 1 mM MgCl_2_, 0.5 mM CaCl_2_, 10 mM HEPES, 10 mM EGTA, 3 mM MgATP, and 0.3 mM NaGTP– (pH 7.20). Electrode tips were coated with Sylgard 184 (Dow Corning). Neural activity was measured in the cell-attached voltage-clamp configuration with holding potential matched to the resting membrane potential (V_hold_ = −60 mV) and with no current generated by the amplifier (*I*_amp_ = 0 pA). Firing rate histograms were generated by integrating action potential discharge in 10- to 20-s bins using Spike 5.0 software (Cambridge Electronic Design, CED). Whole-cell voltage-clamp (V_hold_ = –60 mV) recordings were made to characterize effects of adenosine on intrinsic cellular and synaptic mechanisms. In the presence of tetrodotoxin (TTX; 0.5 μM) to block neuronal action potentials, we followed the time course of CO_2_/H^+^- and adenosine-induced changes in holding current and conductance by delivering intermittent (0.2 Hz) voltage steps (–100 mV). Steady-state current−voltage (*I-V*) relationships were obtained using voltage steps between –40 and –130 mV (Δ 10 mV) under each experimental condition; CO_2_/H^+^-sensitive and adenosine-sensitive difference currents were determined by digital subtraction and averaged for presentation. Synaptic currents were characterized in the absence of TTX using a Cs^+^-based pipette solution containing the following: 135 mM CsCH_3_SO_3_, 10 mM HEPES, 1 mM EGTA, 1 mM MgCl_2_, 3.2 mM TEA-Cl, 5 mM Na-phosphocreatine, 4 mM MgATP, and 0.3 mM NaGTP. To record spontaneous IPSCs (sIPSCs), cells were held at the reversal potential for AMPA-mediated excitatory synaptic currents (sEPSCs; Ihold = 0 mV) and confirmed with bath application of GABA and glycine blockers. IPSC reversal was obtained under bath application of kynurenic acid at 1- to 5-min interval voltage steps ranging from 50 to –20 mv. EPSCs were recorded at a holding potential of the measured IPSC reversal of –60 mv and confirmed with bath application of 6-cyano-7-nitroquinoxaline-2,3-dione (CNQX). Spontaneous EPSCs and IPSCs were analyzed using the Mini Analysis Program (Synaptosoft) and detected events based on amplitude (minimum 5 pA) and characteristic kinetics (fast rising phase followed by a slow decay). Each automatically detected event was also visually inspected to exclude obvious false responses. All whole-cell recordings had an access resistance (Ra) < 20 MΩ, recordings were discarded if Ra varied >10% during an experiment, and capacitance and Ra compensation (70%) were used to minimize voltage errors. A liquid junction potential of –10 mV (KCH_3_SO_3_) or +11 mV (CsCH_3_SO_3_) was corrected off-line.

### Drugs

TTX (0.5 μM) was purchased from Alomone Labs; all other chemicals were obtained from Sigma, unless otherwise stated. Drugs were bath applied at the following concentrations: adenosine (1 µM) to activate adenosine receptors; 8-phenyltheophylline (8PT; 10 μM) to non-selectively block adenosine receptors; 8-cyclopentyl-1,3-dipropylxanthine (DPCPX; 30 nM) to selectively block A1 receptors; CNQX (10 μM) to block AMPA receptors; kynurenic acid (1 mM) to block ionotropic glutamate receptors; strychnine (2 μM) to block glycine receptors; gabazine (10 μM) or bicuculline (10 µM) to block GABA_A_ receptors; tetraethylammonium (TEA; 10 mM) and 4-aminopyridine (4AP, 50 µM) to block voltage-dependent K^+^ channels; and Ba^2+^ (100 µM) to block inward rectifying K^+^ channels.

### Data analysis

Data are reported as mean ± SEM. All statistical analyses were performed in GraphPad Prism 7 (GraphPad Software, Inc.). Data were normally distributed (Shapiro–Wilk normality test), and comparisons were made using *t* test or two-way repeated measure ANOVA followed by Tukey or Dunnett multiple comparison test as appropriate. Relevant values used for statistical analysis are included in the results section as follows: (*t* test) *t* subscript degrees of freedom = *t* statistic; (ANOVA) *F* subscript between-groups degrees of freedom, within groups degrees of freedom = *F* statistic.

## Results

We characterized the effects of adenosine on a total of 66 chemosensitive RTN neurons isolated from the same number of pups (we typically isolate two RTN slices per animal and obtain a successful recording with ∼50% efficiency) from 30 different litters. These cells were functionally identified based on their firing response to CO_2_/H^+^. Neurons were considered chemosensitive if they were spontaneously active under control conditions (5% CO_2_ and 26 mM HCO_3_^–^; pHo 7.3) and responded to 15% CO_2_ (pHo ∼6.9) with at least 1.5-Hz increase in firing. Note that RTN neurons exhibit a linear firing response over this pH range (Guyenet et al., 2005) and all cells included in this study exhibited a CO_2_/H^+^ response profile similar to what has been reported for rat pups (Wenker et al., 2012; Hawkins et al., 2015) and type 1 chemoreceptors in mice (Lazarenko et al., 2009; Wang et al., 2013). Neurons that showed <1.5-Hz firing response to 15% CO_2_ were considered non-chemosensitive.

**Figure 1.**
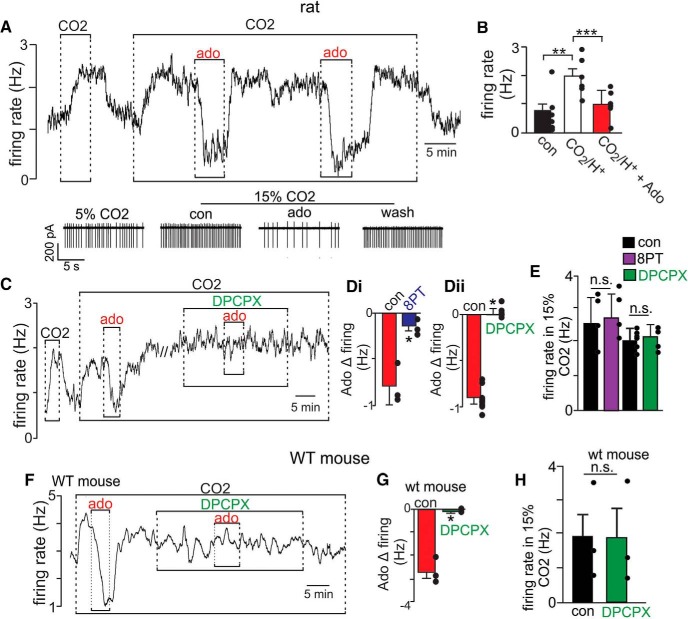
Adenosine strongly inhibits chemosensitive RTN neurons in slices from rat and mouse pups by an A1 receptor-dependent mechanism. ***A***, Trace of firing rate (Hz) and segments of holding current from a chemosensitive neuron in a brainstem slice from a rat pup shows that exposure to adenosine (1 µM; ado) decreases activity in a reversible and repeatable manner. ***B***, Summary data (*n* = 6) shows ado inhibits CO_2_/H^+^ (15% CO_2_)-stimulated activity. ***C***, Trace of firing rate from a chemosensitive RTN neuron in a brainstem slice from a rat pup shows that bath application of a selective A1 receptor blocker (DPCPX, 30 nM) had negligible effect on CO_2_/H^+^-stimulated activity but completely eliminated the inhibitory effects of ado (1 µM). ***D***, Summary data shows the inhibitory effects of ado RTN chemoreceptors in slices from rat pups was eliminated by preincubation (∼10 min) in a non-specific ado receptor blocker (8PT, 10 µM; *N* = 3; ***Di***) or a selective A1 receptor blocker (DPCPX, 30 nM; *N* = 5; ***Dii***). ***E***, Summary data shows bath application of 8PT or DPCPX alone minimally affected CO_2_/H^+^-stimulated activity of RTN chemoreceptors in slices from rat pups, suggesting endogenous ado does not limit chemoreceptor activity under these experimental conditions. ***F***, Firing rate (Hz) trace from a chemosensitive RTN neuron in a brainstem slice from a mouse pup shows that exposure to ado (1 µM) inhibited CO_2_/H^+^-stimulated activity under control conditions but not in the presence of DPCPX (30 nM). ***G***, ***H***, Summary data (*N* = 3) shows in mouse that DPCPX blocked the effect of adenosine, and when applied alone did not affect the CO_2_/H^+^-stimulated activity; *p < 0.05; **p < 0.01; ***p < 0.001.

To determine whether and how adenosine modulates activity of chemosensitive RTN neurons, we first characterized the effects of adenosine on CO_2_/H^+^ sensitivity of RTN neurons in slices from rat pups under control conditions and in the presence of adenosine receptor blockers. Based on our previous results suggesting adenosine blunts CO_2_/H^+^-dependent output of the RTN in adult rats (Falquetto et al., 2018), we expected adenosine to also be inhibitory at the cellular level, and since chemosensitive neurons have low basal activity (0.7 ± 0.3 Hz, *N* = 6), we tested adenosine on CO_2_/H^+^-stimulated activity. We found that bath application of adenosine (1 μM) decreased chemoreceptor activity by 1.2 ± 0.17 Hz (*t*_(9)_ = 6.923, *p* < 0.0001), and it did so in a reversible and repeatable manner (Fig. 1*A*,*B*). Although not systematically tested, we also found that neurons that did not respond to CO_2_/H^+^ also did not respond to adenosine (adenosine Δ in activity 0.1 ± 0.02 Hz; *t*_(2)_ = 3.291, *p* = 0.081). To control for potential CO_2_/H^+^-dependent effects, we confirmed that adenosine also inhibits neural activity at a control CO_2_ level of 5% but with neural activity increased 1.5 Hz by DC current injection (*t*_(3)_ = 12.13, *p* < 0.01). Consistent with our recent *in vivo* results from adult rats (Falquetto et al., 2018), we also found the inhibitory effects of adenosine on chemoreceptor activity was largely eliminated by bath application of a broad spectrum adenosine receptor blocker (8PT; 10 μM; *t*_(2)_ = 4.528, *p* < 0.05) or a selective A1 receptor blocker (DPCPX; 30 nM; *t*_(4)_ = 6.964, *p* < 0.01; Fig. 1*C*,*D*). However, contrary to our *in vivo* results (Falquetto et al., 2018), neither 8PT (*t*_(4)_ = 1.34, *p* = 0.2514) nor DPCPX (*t*_(4)_ =0.9284, *p* = 0.4057) alone potentiated the firing response to CO_2_ (Fig. 1*E*), suggesting endogenous adenosine does not limit CO_2_/H^+^ sensitivity of RTN neurons under these experimental conditions.

Based on evidence for species differences in A1 receptor modulation of respiratory activity at the level of the pre-Bötzinger complex (Zwicker et al., 2011), we also wanted to determine whether adenosine inhibits RTN chemoreceptor activity by an A1 receptor-dependent mechanism in slices from wild-type mice. We found that adenosine (1 μM) also inhibited activity of RTN neurons by an A1-dependent mechanism (*t*_(5)_ = 6.489, *p* < 0.01; Fig. 1*F*,*G*). Also, consistent with evidence that respiratory rhythm generation by the pre-Bötzinger complex is more sensitive to A1 receptor inhibition in mice compared to rat (Zwicker et al., 2011), we found that adenosine inhibition of chemoreceptor activity was more pronounced in mice than rat (*t*_(15)_ = 2.253, *p* < 0.05; Fig. 1*D*,*G*). However, despite the more robust response to adenosine, these cells showed minimal change in activity during exposure to DPCPX alone (*t*_(4)_ = 0.9284, *p* = 0.4057; Fig. 1*H*). These results are consistent with evidence from rat and so further suggest that in both species endogenous adenosine does not limit CO_2_/H^+^ sensitivity of RTN neurons under these experimental conditions. We also did not observe residual responses to adenosine in the presence of DPCPX, suggesting blockade of A1 receptors did not reveal roles for A2 or A3 receptors that were otherwise masked by A1 receptor inhibition. Together, these results identify A1 receptors as the primary target for adenosine modulation of chemosensitive RTN neurons in both rats and mice. For consistency with our previous *in vivo* study in rats (Falquetto et al., 2018), we chose to use this species (rat) for all subsequent cellular experiments to characterize mechanisms contributing to A1 inhibition of RTN neurons.

### Adenosine/A1 receptor signaling activates a GIRK-like conductance in chemosensitive RTN neurons

**Figure 2.**
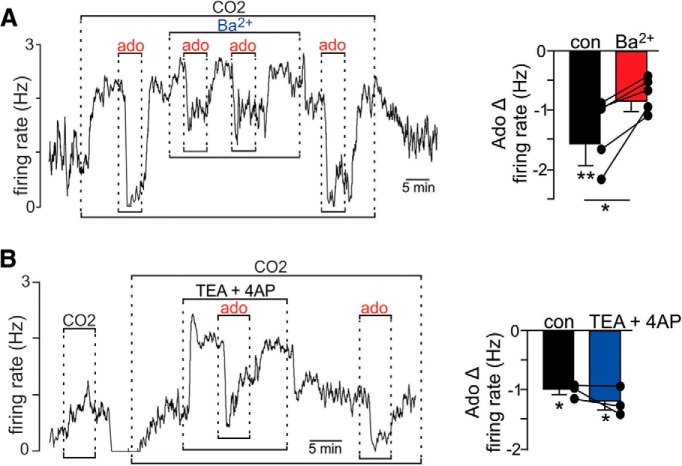
Adenosine inhibition of RTN chemoreceptor activity was blunted by barium. ***A***, Trace of firing rate (Hz) from a chemosensitive RTN neuron in a brainstem slice from a rat pup shows that exposure to adenosine (1 µM; ado) alone caused a near complete suppression of CO_2_/H^+^ (15% CO_2_)-stimulated activity. Application of Ba^2+^ (100 μM) in the continued presence of high CO_2_ caused a modest increase in neuronal activity and blunted subsequent responses to adenosine. Summary data (*N* = 5) to the right show that 100 μM Ba^2+^ blunted the inhibitory effect of ado by ∼50%. ***B***, Trace of firing rate (Hz) and summary data to the right show that ado (1 µM) inhibition of RTN chemoreceptor activity was retained in the presence of TEA (10 mM) and 4AP (50 µM). These results suggest that ado inhibits activity of RTN chemoreceptors by activation of an inward rectifying K^+^ channel; *p < 0.05; **p < 0.01.

To identify mechanisms contributing to A1 receptor inhibition of RTN neurons, we initially focused on postsynaptic mechanisms involving activation of K^+^ channels. Since GIRK channels are a primary downstream target of A1 receptors (Lüscher et al., 1997; Cunha, 2001), we tested the effects of adenosine on RTN chemoreceptor activity in the presence of high CO_2_ before and after bath application of Ba^2+^ to block inward rectifying K^+^ channels including GIRK (Kim and Johnston, 2015). Exposure to Ba^2+^ (100 μM) in the presence of 15% CO_2_ increased neural activity by 0.445 ± 0.15 Hz (*t*_(4)_ = 2.891, *p* < 0.05). Note that Ba^2+^ will also block astrocyte Kir4.1 channels leading to astrocyte depolarization (Wenker et al., 2010) which may also stimulate chemoreceptor activity by facilitating ATP release (Wenker et al., 2010; Sobrinho et al., 2017) or by limiting astrocyte uptake of K^+^ or glutamate (Olsen et al., 2015). In any case, in the continued presence of Ba^2+^ and high CO_2_ subsequent application of adenosine (1 μM) decreased neural activity by 0.73 ± 0.12 Hz (*t*_(4)_ = 3.36, *p* < 0.05; Fig. 2*A*), reflecting a 56% inhibition which is smaller than the adenosine response before addition of Ba^2+^ and after washing Ba^2+^. To rule out potential involvement of voltage-dependent outward rectifying K^+^ channels, we also tested effects of adenosine in the presence of TEA and 4AP. We found that application of TEA (10 mM) and 4AP (50 µM) during exposure to high CO_2_ increased neural activity (0.60 ± 0.28 Hz) but minimally affected the inhibitory response to adenosine (*t*_(2)_ = 1.396, *p* = 0.2975; Fig. 2*B*).

**Figure 3.**
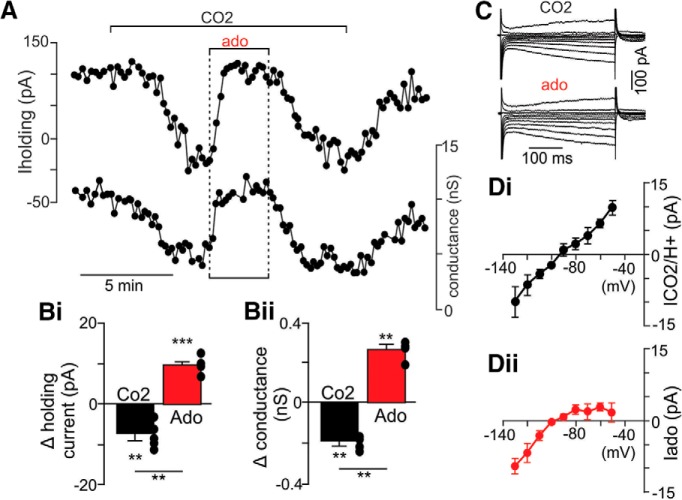
Adenosine activates an inward rectifying K^+^ conductance in chemosensitive RTN neurons. ***A***, Traces of holding current (top) and conductance (bottom; V_hold_ = –60 mV; TTX) from a chemosensitive RTN neuron in a brainstem slice from a rat pup shows that exposure to 15% CO_2_ decreased outward current and conductance. In the continued presence of high CO_2_, subsequent exposure to ado (1 µM) increased outward current and conductance. ***B***, Summary data (*N* = 5) shows the effects of high CO_2_ alone and CO_2_ plus ado on holding current (***Bi***) and conductance (***Bii***). ***C***, Current responses to voltage steps from –60 mV to between –40 and –130 mV during exposure to high CO_2_ alone and CO_2_ plus ado. ***D***, Average (*N* = 5) current–voltage (*I–V*) relationships of the CO_2_/H^+^-sensitive (***Di***) and ado-sensitive (***Dii***) currents; difference currents were isolated by subtracting *I-V* relationships recording during exposure to 15% CO_2_ or ado from those recorded under control conditions; **p < 0.01.

We also characterized the voltage-dependent properties of the adenosine-sensitive current in chemosensitive RTN neurons. These experiments were performed in whole-cell voltage-clamp mode (V_hold_ = –60 mV; 0.5 µM TTX) under similar high CO_2_ conditions as described above. Consistent with previous evidence (Mulkey et al., 2004; Kumar et al., 2015; Guyenet et al., 2016), exposure to high CO_2_ decreased outward current and conductance (Fig. 3*A*,*B*) by inhibition of a voltage-independent K^+^ conductance (Fig. 3*C*,*Di*). Once the CO_2_/H^+^ response stabilized, subsequent addition of adenosine (1 µM) to the bathing medium increased outward current and conductance by 17.5 ± 2.1 pA and 0.5 ± 0.1 nS, respectively (Fig. 3*A*,*B*). The adenosine-sensitive current (i.e., difference current) reversed near the reversal potential for K^+^ (EK) and showed strong inward rectification at voltage positive to EK (Fig. 3*C*,*Dii*). These results show that adenosine activates a K^+^ conductance in chemosensitive RTN neurons with a voltage and pharmacological profile reminiscent of GIRK channels.

### Adenosine/A1 receptor signaling preferentially inhibits excitatory synaptic input to chemosensitive RTN neurons

**Figure 4.**
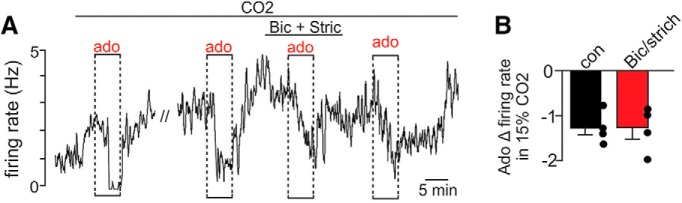
Adenosine modulation of RTN chemoreceptors was retained when GABA and glycine receptors are blocked. ***A***, ***B***, firing rate trace from a chemosensitive RTN neuron (***A***) and summary data (***B***; *N* = 4) shows the inhibitory effects of adenosine (1 µM) are retained in the presence of bicuculline and strychnine.

To determine whether adenosine inhibition of RTN neurons also involves a presynaptic mechanism, we exposed neurons to repeated bouts of adenosine, first under control conditions, and then in the presence of inhibitory receptor blockers. As before, under high CO_2_ conditions, bath application of adenosine (1 μM) decreased firing rate by 1.2 ± 0.1 Hz. Exposure to bicuculline (10 μM) and strychnine (2 μM) to block GABA_A_ and glycine receptors, respectively, minimally affected CO_2_/H^+^-stimulated activity (*t*_(3)_ = 1.204, *p* = 0.315) or the firing response to adenosine (*t*_(3)_ = 0.8955, *p* = 0.4365; Fig. 4*A*,*B*). These results suggest adenosine-mediated inhibition of RTN neurons does not involve potentiation of inhibitory synaptic drive.

**Figure 5.**
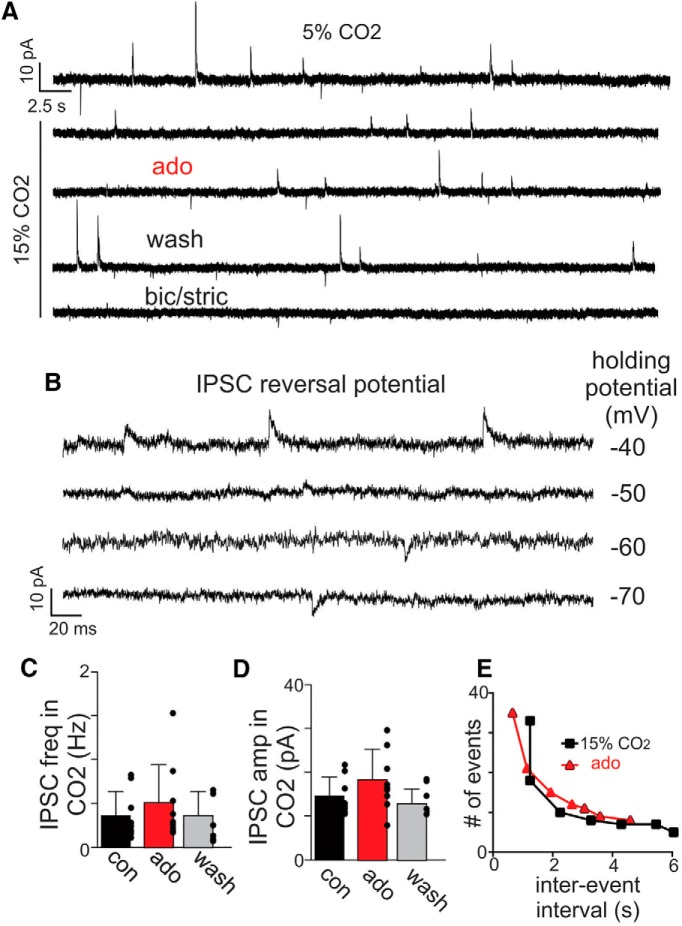
Adenosine minimally affects inhibitory synaptic input to RTN chemoreceptors. ***A***, Traces of holding current (Ihold = 0 mV) from a RTN chemoreceptor shows sIPSC events under control conditions and during 15% CO_2_ alone and with adenosine or bicuculline and strychnine. ***B***, Traces of holding current at holding potentials ranging from –40 to –80 mV (Δ 10-mV steps) and in the presence of kynurenic acid (block glutamate receptors) show that sIPSCs reverse near –60 mV. This holding potential will be used to isolate EPSCs. ***C***, ***D***, Summary data show effects of ado (1 µM) on IPSC freq (***C***) and amplitude (***D***) during high CO_2_. ***E***, Plot of IPSC events versus interevent interval shows ado minimally affected the occurrence of IPSCs.

To further explore this possibility, we characterized the effects of adenosine on inhibitory and excitatory synaptic input to RTN neurons. As above, chemosensitive neurons were identified in cell-attached voltage-clamp mode by their firing response to CO_2_. Once the cell type of interest has been identified, we obtained whole-cell access and in voltage-clamp, recorded spontaneous synaptic currents. sIPSCs were recorded in relative isolation by holding cells at the reversal potential for AMPA-mediated EPSCs (sEPSCs; Ihold = 0 mV). Under control conditions (5% CO_2_) chemosensitive RTN neurons showed sIPSCs with an average frequency of 0.29 Hz and amplitude of 17.61 pA (Fig. 5*A–D*). Bath application of adenosine (1 µM) had negligible effect on sIPSC amplitude (*t*_(8)_ = 1.586, *p* = 0.1514) and frequency (*t*_(8)_ = 1.79, *p* = 0.1113; Fig. 5*A*,*C–E*). In separate experiments, we determined that sIPSCs measured in RTN neurons have a reversal potential near the Cl^–^ chloride reversal potential of –57 mV (based on internal and external Cl^–^ concentrations of 15.2 mM and 141 mM, respectively) and were blocked by bath application of bicuculline (10 µM) and strychnine (20 µM), thus confirming they are mediated by GABA or glycinergic input (Fig. 5*A*,*B*).

**Figure 6.**
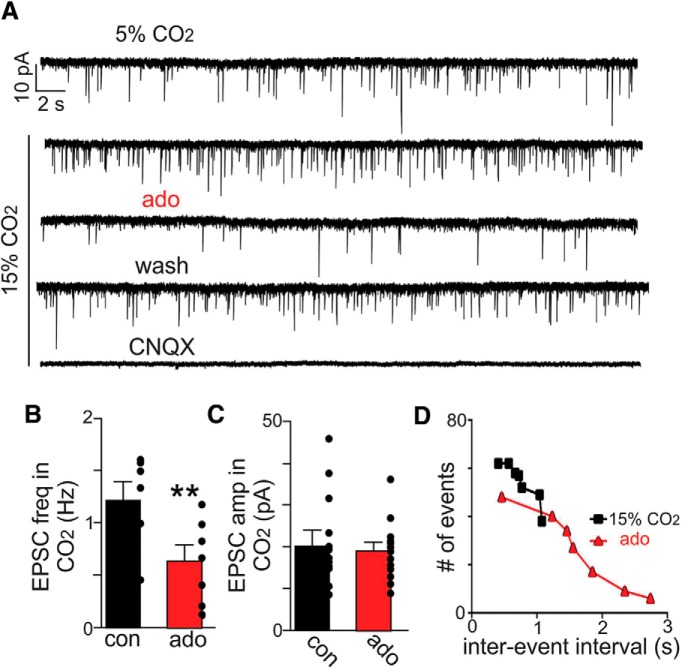
Adenosine decreased excitatory synaptic input to RTN chemoreceptors. ***A***, Traces of holding current (Ihold = –60 mV) from a chemosensitive RTN neuron shows sEPSC events under control conditions and in 15% CO_2_ alone and with ado (1 µM) or CNQX. ***B***, ***C***, Summary data show effects of ado on EPSC frequency (***B***) and amplitude (***C***) during high CO_2_. ***D***, Plot of EPSC events versus interevent interval show that EPSC in ado occurred less frequently. Note that for the dataset shown in ***B***, two outlier data points were identified using ROUT method and excluded from analysis; **p < 0.01.

Next, we measured sEPSCs in chemosensitive RTN neurons (Ihold = –60 mV, near the IPSC reversal potential) during exposure to high CO_2_ and adenosine. We found that exposure to 15% CO_2_ increased frequency but not amplitude of sEPSCs (Fig. 6*A*). This is interesting because it suggests chemosensitive RTN neurons may function as a CO_2_/H^+^-sensing network by forming CO_2_/H^+^-dependent recurrent excitatory connections. Considering extracellular acidification generally suppresses neural excitability and excitatory synaptic transmission (Sinning and Hübner, 2013), we do not think this response results from non-specific effects of H^+^ on neurotransmission. However, this possibility remains speculative since we cannot exclude potential effects of CO_2_/H^+^ on transmitter release from synaptic terminals in the RTN from distal neurons including from other chemosensing regions.

We also found that exposure to adenosine (1 µM) preferentially disrupted excitatory input to chemosensitive RTN neurons; adenosine decreased sEPSC frequency from 2.03 ± 0.54 Hz to 0.85 ± 0.19 Hz (*t*_(6)_ = 4.251, *p* < 0.01; Fig. 6*B*) and expanded the interevent interval (Fig. 6*D*) but with no change in sEPSC amplitude (*t*_(12)_ = 0.6844, *p* = 0.5068; Fig. 6*C*). At the end of each experiment, we bath applied CNQX (10 µM) to confirm sEPSCs are mediated by AMPA receptors (Fig. 6*A*). These results are consistent with evidence from the hippocampus (Lambert and Teyler, 1991; Yoon and Rothman, 1991) and cortex (Qi et al., 2017), suggesting adenosine preferentially disrupts excitatory but not inhibitory synaptic activity.

## Discussion

The main finding of this study is that adenosine and A1 receptor signaling inhibits chemosensitive RTN neurons by mechanisms involving direct neural inhibition by activation of an inward rectifying K^+^ conductance, and by selective suppression of excitatory synaptic input to chemoreceptors. These results are consistent with known mechanisms by which adenosine and A1 receptors inhibits neural activity in other brain regions (Cunha, 2001; Dunwiddie and Masino, 2001), and identify GIRK-like conductance as the first target of purinergic signaling in chemosensitive RTN neurons.

The presumed source of adenosine in the RTN is the rapid breakdown of extracellular ATP (Dunwiddie and Masino, 2001) which has been shown to increase by several μM in the region during exposure to high CO_2_ (Gourine et al., 2005). There is also evidence suggesting that ATP-purinergic signaling through P2 receptors (probably P2Y; Mulkey et al., 2006) enhances CO_2_/H^+^-dependent output of the RTN (Gourine et al., 2010; Wenker et al., 2012; Barna et al., 2016). Therefore, the inhibitory role of adenosine is expected to coincide with and offset the excitatory effects of CO_2_/H^+^-dependent purinergic activation of RTN neurons. This possibility is supported by *in vivo* evidence from adult rats showing that blockade of A1 receptors systemically (Montandon et al., 2007) or in the RTN (Falquetto et al., 2018) potentiated the ventilatory response to CO_2_. We also confirmed that the excitatory P2 receptor-dependent component of this mechanism functions in the reduced slice preparation (Wenker et al., 2010, 2012). However, we show here that A1 receptor blockade by bath application of DPCPX during high CO_2_ failed to potentiate activity of RTN chemoreceptors in slices from neonatal rats or mice, suggesting that endogenous adenosine does not limit the firing response of chemoreceptors under these reduced experimental conditions.

A number of factors may contribute to this apparent lack of endogenous A1 signaling *in vitro*. In particular, solutions used for brain slice experiments are equilibrated with a high O_2_ gas mixture resulting in bath O_2_ levels ∼10-fold higher than *in vivo* (Mulkey et al., 2001) and enhanced superoxide production (D'Agostino et al., 2007). This is relevant because oxidative stress including superoxide has been shown to decrease the activity of ecto-5’-nucleotidase (the enzyme responsible dephosphorylating AMP to adenosine) and limit adenosine production (Obata and Nakashima, 2017). Therefore, it is possible that supraphysiological O_2_ levels used for brain slice experiments precluded adenosine production and A1 signaling. It is also important to recognize that ventilatory suppression by adenosine *in vivo* may involve vasodilation and subsequent washout of tissue CO_2_/H^+^ (Hawkins et al., 2017), and since brain slices lack vascular smooth muscle tone, this inhibitory role of adenosine would not be evident in slices. Although animals of different ages were used for *in vivo* (adult) and *in vitro* (neonatal) experiments, we do not think this contributed to the lack of A1 inhibition *in vitro* since respiratory suppression by adenosine and A1 receptors tends to be more pronounced at earlier developmental time point (Cunha, 2001; Montandon et al., 2008; Funk, 2013). Other factors such as tissue damage incurred during slicing or our preference to test adenosine during exposure to high CO_2_ are also unlikely factors since ectonucleotidase activity and adenosine levels typically increase in response to trauma (Burnstock, 2017) and are stable over the pH range encompassed by our study (Zimmermann et al., 2012). Based on the above caveats, we do not exclude the possibility that endogenous adenosine signaling through A1 receptors limits CO_2_/H^+^-stimulated activity of RTN chemoreceptors.

In many brain regions, adenosine signaling through A1 receptors inhibits neural activity by both presynaptic and postsynaptic mechanisms. We identified a similar paradigm in the RTN; under experimental conditions designed to isolate postsynaptic mechanisms (in TTX to block neural action potentials), we found that exposure to adenosine activated an outward K^+^ conductance reminiscent of GIRK channels; the adenosine-sensitive current reversed near EK and showed strong inward rectification at voltages positive to approximately –90 mV. Also, consistent with evidence suggesting intrinsic CO_2_/H^+^ sensing by RTN neurons involves inhibition of one or more background K^+^ channels (Guyenet and Bayliss, 2015; Kumar et al., 2015; Guyenet et al., 2016), we found that exposure to high CO_2_/H^+^ decreased outward current and conductance by inhibition of a voltage-independent K^+^ current. Importantly, the adenosine -sensitive current was retained during exposure to high CO_2_, thus allowing this mechanism to temper CO_2_/H^+^-stimulated chemoreceptor activity. In addition to activation of GIRK channels, A1 receptors have also been shown to inhibit neural activity by cAMP-dependent inhibition of hyperpolarization-activated cyclic nucleotide-gated (HCN) channels (Li et al., 2011). Although HCN channels regulate basal activity of RTN chemoreceptors (Hawkins et al., 2015), based on the pharmacology (Fig. 2) and voltage-dependent properties of the adenosine -sensitive current (Fig. 3), we do not think HCN channels are targets of A1 signaling in the RTN. HCN channels are also inhibited by depolarization (Biel et al., 2009) and do not contribute to CO_2_/H^+^ sensitivity of RTN neurons (Hawkins et al., 2015; Mulkey et al., 2015); therefore, the preferential A1 targeting of GIRK channels but not HCN channels is consistent with the role of A1 receptors serving as a brake to CO_2_/H^+^-stimulated activity.

At the network level, we find that adenosine and A1 signaling preferentially suppressed spontaneous excitatory but not inhibitory synaptic input to chemosensitive RTN neurons. All EPSCs were eliminated by bath application of CNQX, confirming they are mediated by glutamatergic AMPA receptors. These results are consistent with evidence from the hippocampus (Lambert and Teyler, 1991; Yoon and Rothman, 1991) and cortex (Qi et al., 2017) and suggest presynaptic A1 receptors or the machinery associated with suppression of transmitter release are preferentially expressed in excitatory terminals. Previous work has shown that inhibitory neurons within the ventrolateral medulla in the vicinity of the RTN express A2 receptors (Zaidi et al., 2006) and systemic application of an A2 receptor agonist has been shown to suppress breathing by a GABAergic-dependent mechanism (Thomas and Spyer, 1999; Wilson et al., 2004). Although this A2-dependent mechanism may suppress respiratory activity at other levels of the circuit, and perhaps contribute to the respiratory suppression observed in A1R^-/-^ mice, we do not think this mechanism contributes to adenosine modulation of the RTN since adenosine minimally affected inhibitory input to chemosensitive neurons.

In sum, adenosine is a potent modulator of respiratory function including RTN chemoreception where activation of A1 receptors has been shown to inhibit RTN chemoreceptor function adult rats and in slices from neonatal rats and mice. Mechanisms contributing to A1 inhibition of RTN neurons involve activation of a GIRK-like K^+^ conductance and suppression of excitatory synaptic input. These results may be clinically relevant since they identify chemosensitive RTN neurons as candidates for the respiratory-stimulating effects of caffeine (D'Urzo et al., 1990; Pianosi et al., 1994), an A1 and A2 receptor antagonist used therapeutically to mitigate breathing problems in premature infants (Stevenson, 2007).

## Synthesis

Reviewing Editor: Quentin Pittman, University of Calgary Decisions are customarily a result of the Reviewing Editor and the peer reviewers coming together and discussing their recommendations until a consensus is reached. When revisions are invited, a fact-based synthesis statement explaining their decision and outlining what is needed to prepare a revision will be listed below. The following reviewer(s) agreed to reveal their identity: Patrice Guyenet. Note: If this manuscript was transferred from JNeurosci and a decision was made to accept the manuscript without peer review, a brief statement to this effect will instead be what is listed below.

One of the original J Neuroscience reviewers has returned a review of your submission and your revisions. While the reviewer has continued concerns about novelty, we have agreed that the paper is now close to being suitable for publication. However, there are a number of comments, largely editorial in nature that you must deal with; these are appended below.

In addition, I have concerns about litter effects. You have a very respectable 'n', but it is important to know from how many litters each set of data were obtained to ensure that the data do not suffer from litter effects. Please provide this information either in the methods or in reporting the results.

Here are the editorial comments:

1. Introduction line 148:

“In addition, to gain further insight into roles of endogenous adenosine A1 signaling in control of breathing, we also characterize baseline breathing and the CO2/H+ dependent drive to breathe in A1 receptor knockout mice”. These experiments are no longer presented in this version of the study. This seems to be a left-over from the prior submission.

2. Line 134 “Adenosine A1 receptors are Gi/Go-coupled and in other brain regions known to inhibit neural” Please rephrase: the sentence reads as if brain regions as opposed to A1 receptors inhibit neural activity.

3. The phrase “at the network level” does not add anything and is used over and over. Please consider eliminating it.

4. Line 334 “before and after washing” barium. Rephrase. “Before washing' would be while barium is present. I presume that you mean ”before addition of barium and after washing the barium.

5. Lines 380 to 385. Did you check whether acidification actually suppresses neural excitability and excitatory synaptic transmission in the brainstem in neurons other than RTN?

6. Line 418. What is a P2 component? purinergic receptor?

7. Line 440: encured?

8. Lines 227-242. This paragraph also seems a left-over from a prior version of the manuscript? No plethysmography results are reported in the present iteration.
